# The Effect of Short-Wavelength White LED Illumination throughout the Night on the Milk Fatty Acid Profile of High-Yielding Dairy Cows

**DOI:** 10.3390/biology11121799

**Published:** 2022-12-11

**Authors:** Aviv Asher, Matan Fialko, Florin Fares, Uzi Moallem, Shamai Yaacoby, Roee Gutman

**Affiliations:** 1 Northern R&D, MIGAL–Galilee Research Institute, P.O. Box 831, Kiryat Shmona 11016, Israel; 2 Department of Animal Sciences, Faculty of Sciences and Technology, Tel-Hai College, Upper Galilee 12210, Israel; 3 Laboratory of Integrative Physiology, The Department of Nutrition and Natural Products, MIGAL–Galilee Research Institute, Kiryat Shmona 11016, Israel; 4 Agricultural Research Organization, Department of Ruminant Science, P.O. Box 15159, Rishon LeZion 7528809, Israel

**Keywords:** photoperiod, cow, day milk, night milk, circadian rhythm, milk fat, monounsaturated fatty acids, saturated fatty acids, fat composition

## Abstract

**Simple Summary:**

Exposure to artificial white light at night (LAN) disrupts circadian rhythms, yet many dairy cows continue to be exposed to LAN for historical and practical reasons. We explored the effect of whole-night illumination, using white light-emitting diode (LED) bulbs, on the production and composition of milk collected during the day (day milk) and night (night milk). Compared to a natural light–dark cycle, white LED reduced the differences in milk composition between milking hours, yet did not affect milk production. Specifically, white LED increased the percentage of saturated fatty acids in day milk at the expense of unsaturated fatty acids. Therefore, we conclude that natural light is preferable to white LED for cows’ well-being and for preserving the natural milk fat profile. Maintaining the natural milk fat profile results in obtaining day milk fat with higher levels of unsaturated fatty acids, mainly oleic acid, which are known for their health-promoting characteristics. In conclusion, reducing the exposure of dairy cows to white LED during the night will reduce energy use in dairy farms, save electricity costs, and yield day milk with a healthier fat composition, and possibly a higher health-promoting market value. The welfare conditions of the cows will also improve.

**Abstract:**

Fatty acid levels in milk vary between day and night milking. Many dairy cows are still kept under white light-emitting diode (W-LED) illumination throughout the night, although it is known to disrupt endogenous circadian rhythms. We investigated the effects of whole-night W-LED illumination (125 lux) on milk yield and circadian composition, compared to a natural light–dark (LD) cycle of 10 h light. Mid–late lactation cows (*n* = 34) that were exposed to natural LD cycle showed circadian variation in milk fat composition, characterized by higher health-promoting monounsaturated fatty acid (MUFA; 24.2 ± 0.4 vs. 23.2 ± 0.4 g/100 g fat, *p* < 0.001) and lower saturated fatty acid levels (71.2 ± 0.4 vs. 72.5 ± 0.4, *p* < 0.001) at 13:30 h (day milk) than at 03:30 h (night milk). Compared to natural LD (*n* = 16), W-LED (*n* = 18) did not affect milk production or milk fat yields, yet abolished the milking time variation in milk fat composition towards a less healthy fatty acid profile. This lowered MUFA levels of day milk (23.8 ± 0.4 vs. 26.7 ± 0.4, *p* < 0.01). Therefore, W-LED has no commercial advantage over the tested natural LD cycle, and conversely, even shows circadian disruption. Accordingly, a natural LD cycle of 10 h light is preferable over W-LED from the perspective of cost savings, the cows’ well-being, and preserving the natural milk fat profile, as the nutritional value of the day milk is slightly higher.

## 1. Introduction

Long-term records of dairy milk yield and composition (e.g., fat and protein level) show a cosine-like monthly variation. This is characterized by milk yield peaking around the vernal equinox (March to April), and fat and protein concentrations peaking earlier, around the winter solstice (November to January) [1,2]. Although changes in diet and environmental temperature may contribute to these variations, the natural change in photoperiod (i.e., the daily change in the hours of illumination) is recognized as the dominant factor that drives these annual changes [1]. In addition, the use of artificial illumination has long been recommended to increase daylight up to 16 or 18 h. This is known to increase milk yield, without affecting fat content, compared to a short day with 8 h of illumination or natural daylight of ca. 9.5 to 14.5 h, regardless of the stage of lactation [3,4,5,6,7]. Notably, compared with the 18 h illumination cycle [8] or a natural photoperiod of 13 h of illumination [9], a maximal 24 h illumination cycle does not further increase or decrease milk yield, or protein and fat content, in Holstein cows.

Despite the above, 24 h illumination may have deleterious effects beyond its not increasing cows’ productivity compared with the long-day illumination cycle [8,10]. Human and animal model studies have revealed the physiological harms of constant illumination (known as light at night, LAN) [11]. These models showed that these LAN-induced impairments are due to disruption of the normal circadian physiological cycle, such as the melatonin cycle, and may be associated with an increased prevalence of metabolic diseases and probably cancer [11,12,13]. Therefore, circadian disruption due to LAN following shift working has been declared a pollutant and suggested as a carcinogen by the World Health Organization [14]. In cows, as in other organisms, behavioral and physiological processes show a daily rhythm, with a ca. 24 h period length (i.e., circadian rhythm) that is affected by the photoperiod [15,16]. Notably, and as found in humans and in animal models, our previous research in cows showed LAN-induced circadian disruption in heart rate and melatonin rhythms [17]. This circadian disruption was manifested by a lower milk melatonin level in both day milk (DM) and night milk (NM), and by a lower within-day difference in milk melatonin level between milking times [17]. Despite these data, many dairy workers illuminate the barn all night for operational comfort, believing that this light regime also leads to higher milk yield, due to higher feed consumption.

Dairy workers not only illuminate the barn throughout the night, but do so using light-emitting diode (LED) bulbs. This type of lighting has gained popularity in the agricultural market due to its longer lifespan, higher illumination efficiency (illumination per watt of electric energy), and the option to control the illumination spectrum, compared to traditional bulbs [18,19]. However, white LED (W-LED) illumination raises several concerns [9,20] due to the domination of W-LED by short wavelengths (i.e., ‘blue’ illumination). The latter enhances activation of the photoreceptors that synchronize the internal master clock, hence resulting in a more significant disturbance in daily rhythms than longer wavelengths (i.e., ‘yellow’ illumination) [9,11,20]. Indeed, LAN using ‘blue’ illumination was found to be more stressogenic to lactating cows than ‘yellow’ illumination [9], and the circadian disrupting effect of ‘blue’ illumination (e.g., suppression of plasma melatonin level vs. ‘yellow’ illumination or red illumination) was even shown when these wavelengths were present only during the last hours of daytime [21,22].

Milk fat is primarily composed of saturated fatty acids (SFA) [23,24], of which high consumption is suggested as a risk factor for cardiovascular diseases (e.g., atherosclerosis), compared to same-level consumption of unsaturated fatty acids (UFA) [25]. However, reducing SFA intake by reducing dairy food consumption may have limited or possible adverse effects [26]. Therefore, it has been an ongoing effort to explore ways to reduce the SFA levels in milk fat while increasing the unsaturated fatty acid (UFA) levels [27,28]. Notably, for some milk constituents (e.g., fat level), a circadian variation in milk fat composition has been found (i.e., a difference between DM (milked at 12:30 or 15:00) and NM (milked at 04:30 or 05:00)) [29,30,31,32]. Therefore, the present study’s first aim was to re-examine, in a controlled manner, the effect of W-LED LAN on dairy cows’ milk production and milk fat yields compared to a light regime of about 10 to 11 h of natural light. In addition, we aimed to examine the circadian variation in fatty acid composition in milk fat (i.e., DM vs. NM) under the natural light regime; and to investigate the effects of W-LED LAN on this circadian variation. We hypothesized that compared to the natural light regime of ca. 10 to 11 h of natural light, whole-night W-LED illumination does not affect cows’ milk yield, yet disrupts the circadian clock, thus changing the between-milking-time difference in milk fat composition.

## 2. Materials and Methods

### 2.1. Animals, Housing Conditions, and the Experimental Protocol

The Israeli Committee for Animal Care and Experimentation (Beit Dagan, Israel) approved all the animal-involving procedures (Volcani file number 72917 IL). All experiments were performed in accordance with relevant guidelines and regulations. The experiments were conducted at the experimental dairy farm in the Volcani Center, Beit Dagan, located in the central district of Israel. We used two groups of Israeli high-yielding Holstein cows, a control group (*n* = 16) and a treatment group (*n* = 18), housed in two separate yards on the same farm. The initial parameters for block subdivision, i.e., age (3.6 ± 1.3 years in both groups), days at milking (control, 175.3 ± 84.6; treatment, 163.3 ± 82.1), body weight, and daily milk yield, did not differ significantly between the groups (Table 1). We chose to study high-yielding multiparous cows in mid–late lactation because this period is characterized by the highest stability in milk yield, feed intake, energy balance, and production efficiency. This profile of highly stable characteristics enables one to examine the effect of the selected manipulation (i.e., W-LED LAN vs. natural light) on these and other parameters, as suggested by our previous work and other studies [33,34,35].

**Figure 1:**
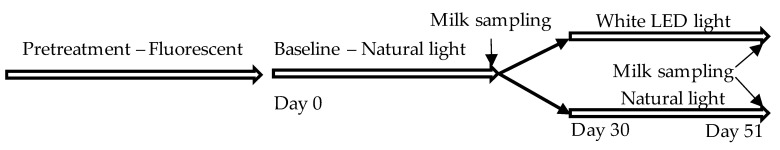
The experimental timeline and sampling.

Cow feed (Table A1) was provided *ad lib* and dispensed daily at 10:00 (or 1 h earlier during daylight savings time, which ended on 27 October 2019, the 30th day of the experiment). During the pre-experimental phase, the yard was illuminated throughout the night using fluorescent bulbs (Figure 1 and Table 1). The cows were regularly milked at 05:00, 13:00, and 20:00 h standard time throughout the pre-experimental phase and all the following experimental phases. During the ca. 0.5 h milking, cows were exposed to white fluorescent illumination (dominant wavelength of 545 nm at 140 lux, measured at cows’ eye height using AvaSpec-2048-FTSDU, Avantes, Eerbeek, Netherlands). As justified above, we examined the response to illumination manipulations in mid–late lactation cows [33,34,35]. Accordingly, the overall length of the experiment was limited to about 60 days, thus avoiding sampling of cows reaching the dry period (Figure 1). We sought to examine the DM vs. NM variation in fatty acid composition under natural illumination, and the effects of W-LED LAN on this variation and on cows’ milk yield. To this end, we first transferred all the cows to a natural light–dark (LD) cycle regime of ca. 11 h light and ca. 13 h dark (Figure 1 and Table 1). The control group was held under this naturally changing LD cycle throughout the experiment, during which the light hours were shortened by ca. 1 h to ca. 10:14 LD cycle (Figure 1 and Table 1). Following 30 days under the natural LD cycle, the illumination conditions of the treatment group were changed to a W-LED illumination regime (dominant wavelength of 462 nm, day light 6500 k, 34 w, Soul LTD, 124.5 Lux). W-LED lights were only turned on throughout the night, resulting in a 24 h illumination regime (24:0 LD cycle, Figure 1 and Table 1).

### 2.2. Sampling of the Milk Fat and Analysis of Its Fatty Acid Profile

Two milk samples were collected 10 h apart, 1–3 days before the end of each of the two experimental phases (Figure 1). One milk sample was taken during the regular milking time of 13:30 (i.e., DM) and another during a novel milking time of 03:30 (i.e., NM). The latter time was set to enable sampling of the milk formed in the mammary gland during the night hours, before sunrise. At each milking event, one 50 mL milk tube from each cow was sent to an external ISO-certified laboratory (Israeli Cattle Breeders’ Association, Caesarea, Israel). There, total fat was analyzed using FTIR (Foss MilkoScan™, Hilleroed, Denmark). Additionally, two 15 mL tubes were sub-sampled and frozen at −20 °C for the fatty acid profile analysis.

Samples were prepared as previously described [36]: First, 0.5 mL of each milk sample was pipetted into a 15 mL tube, followed by 10 mL of hexane (analytical grade, J.T. Baker) spiked with 50 µg/mL of benzophenone (Cat #: 427551, Sigma, Darmstadt, Germany) as an internal standard, and 50 µg/mL tritridecanoate (T3882, Sigma, Darmstadt, Germany) as the transesterification standard. Then, 1 mL of a saturated solution of sodium methoxide (TCI Co., Ltd.) in methanol (HPLC grade, J.T. Baker) was added. The sealed tubes were placed in a rack and vigorously shaken for 20 min while lying horizontally on an orbital shaker. An aliquot of the upper organic layer was filtered through a syringe filter (PTFE filter matrix, 13 mm ⌀, 0.45 µm pore size) directly into a glass amber vile for gas chromatography (GC) analysis. External calibration standards were prepared. First, a standard mix containing 37 fatty acid methyl esters (cat #: 18919, Sigma, Table A2) was dissolved in hexane to a 20 mg/mL concentration and stored in a capped vile as a stock solution at −20 °C. An aliquot from the stock was used to prepare standard dilutions of 4, 3.2, 2.4, 1.6, 0.8, 0.4, and 0.1 mg/mL in hexane. Additionally, a standard of conjugated linoleic acid (CLA, C18:2) and methyl esters (cat #: O5632, Sigma, Darmstadt, Germany) was dissolved in pentane to a 50 mg/mL concentration and stored in a capped vile as a stock solution at −20 °C. An aliquot from this stock was used to prepare standard dilutions of 1, 5, 10, 15, 20, and 25 µg/mL in hexane. Each calibration solution, the standard mix, and the CLA standard contained 50 µg/mL of benzophenone as the internal standard.

The fatty acid profile was analyzed on a GC instrument (7890A, Agilent Tech. Santa Clara, CA, USA) coupled with a mass spectrometer (5975C, Agilent Tech. Santa Clara, CA, USA). Separation was performed on a polar column (30 m length, 0.25 mm internal diameter, Zebron ZB-FAME, Phenominex). The conditions were as follows: First, 1 µL of the sample or calibration standard was injected into the GC inlet, heated to 250 °C, and set to a split ratio of 10:1. The oven program was set to 100 °C for 2 min, then a 10 °C/min increase to 140 °C, then a 3 °C/min increase to 180 °C, ending with a 30 °C/min increase to 260 °C, with another 2 min holding time (the total runtime was 24 min). Fatty acids were quantified as methyl esters using individual calibration curves, as previously described [37]. A calibration curve was plotted with values calculated for each fatty acid: x_i_ = C_i_/C_is_, y_es_ = A_i_/A_is_. Here, X_i_ is the calibrant’s value for a given FAME (plotted on the x-axis); C_i_ is the calibrant concentration for a given FAME as mg/mL; and C_is_ is the standard internal concentration in the calibrant as mg/mL.

### 2.3. Statistical Analysis

Kolmogorov–Smirnov normality tests were applied to assess the normality of the data. As the data were not normally distributed, we used the Mann–Whitney non-parametric test to evaluate differences between the control and treatment groups at each experimental period, and the Wilcoxon non-parametric test to evaluate differences between milking times within each experimental group using SPSS, version 27 (IBM). The significance level was set at *p* < 0.05.

## 3. Results

### 3.1. Milk Fat Composition under the Natural Illumination Regime Is Time-Dependent, Showing Higher Mono- and Polyunsaturated Fatty Acid Levels at Day Milking

**Figure 2:**
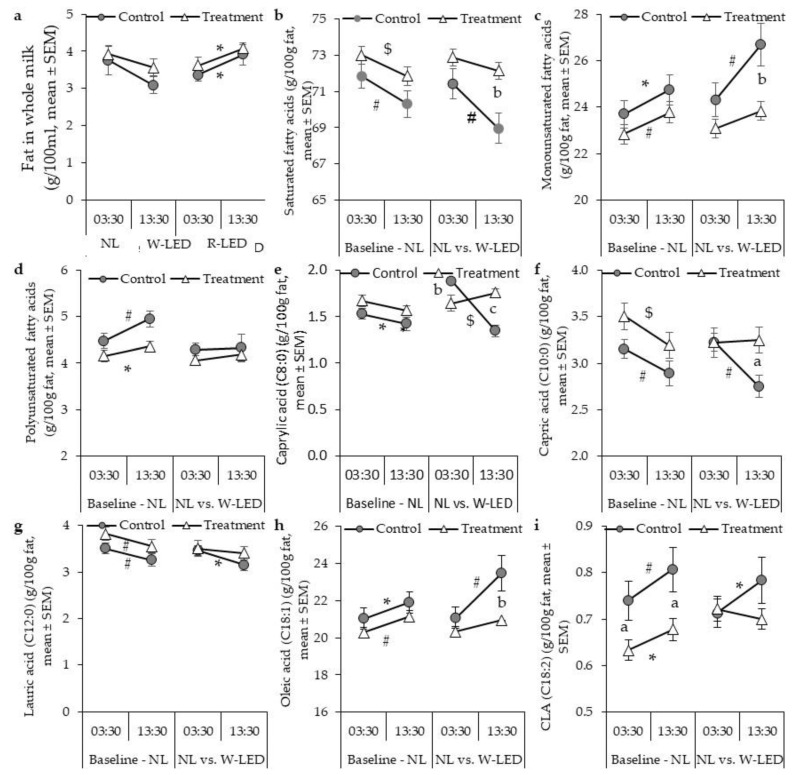
The effects of illumination condition and milking time on milk fat (**a**) and its composition, i.e., the concentration of saturated (**b**), mono (**c**), and polyunsaturated (**d**) fatty acids, and concentration of caprylic (**e**), capric (**f**), lauric (**g**) oleic (**h**) and conjugated linoleic (**i**) acid. The data are presented as mean ± standard error. Control, *n* = 15; treatment, *n* = 18. SFA, saturated fatty acids; MUFA, monounsaturated fatty acids; PUFA, polyunsaturated fatty acids; CLA, conjugated linoleic acid. *, *p* < 0.05; #, *p* < 0.01; $, *p* < 0.001, between milking hours, by Wilcoxon non-parametric test. a, *p* < 0.05; b, *p* < 0.01; c, *p* < 0.001, between treatments within milking hours, by Mann–Whitney non-parametric test.

**Figure 3:**
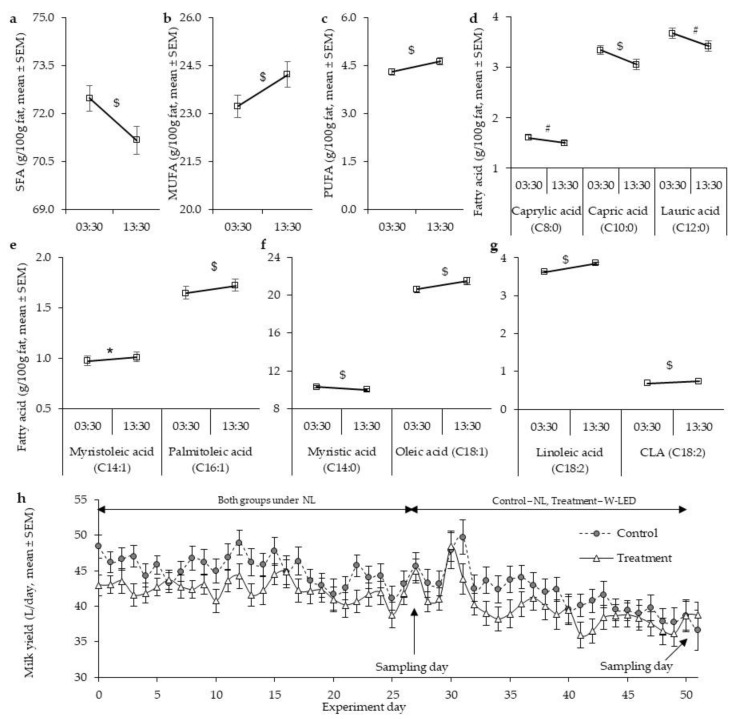
The effects of milking hour on milk fat composition under natural light baseline conditions for cows treated with W-LED and the control group; and the effect of illumination condition on milk yield (**h**). We detected the level of saturated (**a**), monounsaturated (**b**), and polyunsaturated (**c**) fatty acids, caprylic, capric, and lauric fatty acids (**d**), mysritoleic and palmitoleic fatty acids (**e**), myristic and oleic fatty acids (**f**), and linoleic and conjugated linoleic fatty acids (**g**). The data are presented as mean ± standard error. Control, *n* = 15; treatment, *n* = 18. NL, natural light; SFA, saturated fatty acids; MUFA, monounsaturated fatty acids; PUFA, polyunsaturated fatty acids; CLA, conjugated linoleic acid. *, *p* < 0.05; #, *p* < 0.01; $, *p* < 0.001, between milking hours, by Wilcoxon non-parametric test.

Under natural LD cycle baseline conditions, the milking hour affected the concentrations of 9 of the 15 quantified fatty acids (Figure 2 and Table A3). Between-group comparisons showed significant differences only in C18-2 (only in 13:00 milking) and CLA (at both milking hours). These together accounted for only <5% of the total fatty acid content (Table A3). Therefore, we pooled the group LD cycle data (Figure 3). Accordingly, compared to the 03:30 night milking (NM), 13:30 day milking (DM) showed lower SFA levels (71.2 ± 0.4 vs. 72.5 ± 0.4 g/100 g milk fat, *p* < 0.001), and higher levels of monounsaturated fatty acid (MUFA; 24.2 ± 0.4 vs. 23.2 ± 0.4 g/100 g milk fat, *p* < 0.001) and polyunsaturated fatty acid (PUFA; 4.6 ± 0.1 vs. 4.3 ± 0.1 g/100 g milk fat, *p* < 0.001) (Figure 2 and Figure 3, and Table A3). The lower level of SFA at the DM was mainly due to the lower level of short- to mid-chain fatty acids (caprylic (C8:0), capric (C10:0), lauric (C12:0), and myristic acid (C14:0)) (Figure 2 and Figure 3, and Table A3). The higher levels of MUFA and PUFA at the DM were due to the higher levels of all the detected MUFA and PUFA (myristoleic (C14:1), palmitoleic (C16:1), and oleic (C18:1); and linoleic (C18:2) and CLA (C18:2), respectively, (Figure 3 and Table A3)).

Body weight gradually increased throughout the experiment, and milk yield gradually decreased (Figure 3 and Table 1). As a baseline, after another three weeks under natural light conditions, the milking hour still affected most (11 of the 15) quantified fatty acid levels (Figure 2 and Table A3). As was found for baseline milk, SFA levels were lower at DM than NM (69.8 ± 0.8 vs. 71.8 ± 0.8 g/100 g milk fat, *p* = 0.003), while MUFA levels were higher (26.7 ± 0.9 vs. 24.3 ± 0.7 g/100 g milk fat, *p* = 0.003). These differences were due to the same fatty acid that accounted for the corresponding differences measured at baseline (Figure 2 and Table A3). In all, these milking-hour-related differences in milk fat composition, found under natural light conditions of ca. 10–11 illumination hours, are robust. The differences were not eliminated by the combination of a 1 h reduction in the hours of natural light, increased days-at-milking, or decreased milk yield (Figure 2 and Table A3).

### 3.2. Whole-Night White LED Illumination Results in a Higher Level of SFA at DM and Abolishes the between-Milking-Time Variation in Milk Fat Composition Found under the Natural Light Regime

White LED illumination did not affect the cows’ body weight, milk yield, or milk fat levels at each milking hour (Table 1 and Figure 2a,h). W-LED illumination affected the milk fat composition, mainly in DM. This abolished the differences between DM and NM level for eight of the nine fatty acids, and the differences in SFA (72 ± 0.5 and 72 ± 0.8 g/100 g milk fat, respectively, *p* = 0.206) and MUFA (23.8 ± 0.4 and 23.0 ± 0.4 g/100 g milk fat, respectively, *p* = 0.122) (Figure 2 and Table A3). Differences were still observed between DM and NM in control cows: SFA (69.8 ± 0.8 and 71.8 ± 0.8 g/100 g milk fat, respectively, *p* = 0.003) and MUFA (26.7 ± 0.9 and 24.3 ± 0.7 g/100 g milk fat, respectively, *p* = 0.003; Figure 2 and Table A3). This lack of a time-related difference in SFA levels under W-LED was due to the abolishment of time-related differences in butyric (C4:0), caproic (C6:0), caprylic (C8:0), capric (C10:0), lauric (C12:0), margaric (C17:0), and stearic (C18:0) acid levels, which were still present in the control cows, due to their higher levels at DM under W-LED compared to natural light (Figure 2 and Table A3). The complementary effect was found in MUFA levels, which were now lower in the DM of the W-LED treated than in the control cows (Figure 2c and Table A3). This W-LED-induced lower MUFA level at DM was mainly due to a lower oleic acid (C18:1) level than in the control cows (20.9 ± 0.4 vs. 24.0 ± 1.0 g/100 g milk fat, *p* < 0.01); (Figure 2h and Table A3).

## 4. Discussion

In cows and other organisms, many behavioral and physiological outputs show a circadian rhythm that is disrupted by exposure to artificial illumination at otherwise dark hours (i.e., LAN) [11,17]. Our present study shows that exposing high-yield dairy cows to a natural LD cycle of ca. 10–11 h of natural light is accompanied by a circadian variation in milk fat composition. This circadian variation is reflected in higher levels of the health-promoting UFA in DM (milked at 13:30) than in NM (milked at 03:30). This natural between-milking-time variation was abolished under whole-night short-wavelength illumination by W-LED, but 24 h milk yield was not affected. These results suggest that a natural LD cycle of ca. 10–11 h of the natural light regime is preferable over W-LED for cows’ well-being, DM quality, and reducing unnecessary illumination costs.

In dairy cows, as in other organisms, most physiological parameters show a circadian output affected by photoperiod [15,16]. This has prompted investigations of within-day variations in milk composition, to differentiate between DM and NM from health-promoting, nutritional, and commercial perspectives [17,29,30,31,32,38]. As expected, most of these studies found circadian oscillation in milk yield and in some of the milk’s constituents, yet the presence and extent of the circadian variation varied between studies and depended on milking and feeding frequency [17,29,30,31,32,38]. In this study, we mainly focused on the composition of fatty acids, due to their high nutritional value [39]. In cows exposed to natural light conditions, we found 1–2 percentile point higher UFA levels and lower complimentary SFA levels in DM than in NM. This lower level of SFA in DM was due to a difference in short- to mid-chain SFA (C8 to C14). In contrast, the higher level of UFA in DM was due to different levels of all the detected UFA, as found by others [32], mainly oleic acid levels (e.g., a 2.9 percentile point difference at the second time point). These circadian rhythms in milk fat level and composition are suggested to be controlled by the feeding patterns and mammary circadian clock, which synchronizes to the central circadian clock; the latter synchronizes to the photoperiod [16,40]. In all, under natural light conditions of ca. 10–11 illumination hours, our results show a modestly higher nutritional value of DM milk fat, which was richer in UFA compared to NM milk fat. This difference was not affected by the combination of a 1 h reduction in the hours of natural light, increased days-at-milking, or decreased milk yield. Yet, due to the scant nutritional difference between DM and NM, the economic and commercial feasibility is doubtful, regarding the separation of DM from NM milk during milking, and the production of ‘premium milk’ characterized by health-promoting nutritional values.

Our demonstration of a between-milking-time variation in milk fat composition set the ground for exploring our main aim. Specifically, we investigated the effect of whole-night illumination, using W-LED, on milk yield and milk fat composition, compared to a natural illumination regime of about 10 h of natural light. The result of this comparison is of scientific importance for several reasons. For example, although illumination throughout the night does not increase milk production efficiency beyond that achieved under a long-day regime [8,9], some dairy farms still practice it, and, more recently, even use short-wavelength LED illumination of various spectrums and intensities. This usage of LED lighting highlights another important aspect of this study: examining the effect of W-LED LAN per se, which is dominated by short wavelengths, on productivity and the existence of daily endogenic rhythms. Illumination with W-LED showed that whole-night illumination using W-LED bulbs did not affect cows’ body weight, milk yield, or milk fat content; all resembled those of control cows, which continued to be exposed to the natural LD cycle. This corroborates another study that compared outcomes of W-LED LAN to those of a natural photoperiod of 13 h illumination [9]. Therefore, as W-LED LAN, which requires a high energy investment in illumination overnight, did not yield a commercial advantage, we cannot cite any advantage in its usage. It should be noted that we tested the effect of W-LED LAN vs. natural light during the fall. Hence, the study should be repeated under high summer temperatures at which W-LED LAN-exposed cows may shift their feeding hours to the cooler, now-illuminated, “night” hours, hence inducing a more positive energy balance resulting in a higher milk yield. This proposed experiment is also needed as the seminal work showing that a 24 h light regime does not affect milk yield and composition in Holstein cows, compared to an 18L:6D, was conducted during the cold winter time (December to April) rather than in summer time (1), or under comfortable ambient temperature, which characterizes South Korea during the summer and autumn months (2).

Our lack of finding a robust W-LED-induced disruption in body weight and milk yield is somewhat surprising. This contrasts with LAN disruption observed over a range of physiological parameters and circadian rhythms in human and animal models [11], and cows’ circadian rhythm of milk melatonin and heart rate [17]. Hence, we suggest that macro parameters, such as body weight, milk yield, and milk fat level, appear more resistant to a disturbance resulting from W-LED LAN exposure, at least one that lasts for three weeks. This resistance may result from the short, only three-week-long manipulation, or the endless selection of high-yielding cows. The latter favors cows that produce high volumes of milk and milk fat despite their exposure to LAN and its detrimental effects. Nevertheless, the observed ‘resistance’ to the W-LED illumination (spectrum and intensity) used in our study does not infer sweeping resistance to W-LED. This is because W-LED bulbs differ in the composition of their wavelengths. In fact, the dominant wavelength in our W-LED illumination was 462 nm (‘blue’), yet it included other (longer) wavelengths. The usage, however, of W-LED illumination with a higher representation of short wavelength may have resulted in a disruption of such macro parameters as body weight, milk yield, and milk fat, as described in previous work [9].

Despite the above, the lack of a W-LED-induced disruption did not apply to the between-milking-time variation in milk fat composition. Milk and its products are a substantial source of dietary fat in many human populations [41]. However, milk fat comprises a high proportion of SFA [23,24], suggested as a risk factor for cardiovascular diseases (CVD, e.g., atherosclerosis) [42,43]. Nevertheless, reducing SFA consumption by reducing milk consumption may not be the best approach, as milk is rich in essential minerals and amino acids, and milk fat per se is a carrier of fat-soluble vitamins and a source of several essential fatty acids [44]. Both feed-related factors, i.e., dietary intake and seasonal and regional effects, and animal-related factors, i.e., genetics (breed and selection), stage of lactation, mastitis, and ruminal fermentation, are capable of modifying the fatty acid composition, as well as the overall quantity of lipids present in milk [45]. Therefore, manipulating fatty acid composition in milk fat by genetic selection or dietary modification seems beneficial for increasing UFA percentage on account of SFA percentage [28,39].

Another option for obtaining a higher level of UFA in milk fat could be manipulating the photoperiod. Notably, numerous studies searched for an effect of the natural photoperiod, long-day and even whole-night illumination on body weight, milk yield, milk composition, and circadian variation (reviewed above). Moreover, many studies investigated the effect of season-induced change in photoperiod on milk fat composition (e.g., [46,47]). Yet, only a few recent studies investigated circadian variation in milk fat composition and its attenuation by photoperiod [29,32]. Thus, the novelty of this study is our aim of bridging this gap in existing knowledge. Our results show that compared to a natural LD regime, W-LED LAN resulted in higher SFA levels in DM, hence abolishing the between-milking-time variation in SFA and MUFA levels maintained under a natural LD regime in control cows. The elevation in SFA and the circadian disruption were due to elevations in DM levels of butyric (C4:0), caproic (C6:0), caprylic (C8:0), and capric (C10:0) acids compared to control cows. The complementary effect for MUFA levels in DM was mainly due to an impact on the oleic acid (C18:1) level, which was lower in the DM of W-LED-exposed cows than in the control cows. Overall, these results show that W-LED LAN does not affect milk or milk fat yields, but results in a slightly less healthy fatty acid profile, by increasing SFA levels of DM, hence abolishing the between-milking-time variation in milk fat composition. However, as we did not measure the SFA vs. UFA ratio in the milk tank, we cannot evaluate the effect of W-LAN on this parameter. This limitation of the study’s results should be addressed in future studies.

The biochemical mechanisms by which photoperiod, specifically W-LED LAN, affects the milk fat composition of DM are yet to be understood. Notably, the main effect on DM SFA levels was on the less abundant short- to mid-chain (≤C12) fatty acid levels, and not on the most abundant palmitic acid (C-16). This suggests several implications. In contrast to palmitic acid, short- to mid-chain fatty acids are mainly synthesized de novo within the mammary gland rather than partly transferred from the blood to the mammary gland [24]. Therefore, the observed LAN-induced change in DM fatty acid composition may be due to a LAN-induced effect on the within-mammary-gland synthesis of short- to mid-chain fatty acids. Mammary gland cells obtain an endogenous circadian clock and show a circadian rhythm in about 7% of their transcriptome, including core-clock-related and metabolic genes [16]. As these genes are affected by changes in photoperiod [16], we suggest that the LAN-induced changes in DM SFA levels are mainly due to a LAN-induced mammary gland shift in the clock-controlled de novo anabolism of short- to mid-chain fatty acids. Oleic acid originates mainly from feed [24] rather than the mammary gland. Accordingly, the source of the W-LED LAN-induced difference in DM oleic acid level seems external to the mammary gland. Regardless of the source of the changes in milk fatty acid levels, they are probably mediated by the already shown photoperiod-induced changes in feeding patterns [5] that were shown to affect milk levels of both oleic acid and short- to mid-chain fatty acids [40]. The described changes in milk fatty acids are also probably mediated by photoperiod-induced changes in blood melatonin levels, to be measured in future studies, as we already showed that milk melatonin is attenuated by short-wavelength white fluorescent LAN [17].

## 5. Conclusions

We showed no commercial advantage in W-LED LAN compared to the natural LD cycle during the autumn months, at 32° north latitude, consisting of ca. 10 to 11 h light exposure period. However, we showed a disadvantage, namely, a circadian disruption that yielded slightly higher levels in DM, of SFA; increased dietary consumption of the latter has been suggested as a risk factor for metabolic diseases. Hence, our findings support using the natural LD cycle of ca. 10 to 11 h of light over W-LED. This is from the perspective of cost savings, cows’ well-being, and preserving the natural circadian variation in milk fat composition, due to DM’s slightly higher nutritional value.

## Figures and Tables

**Table 1:** Illumination conditions and selected parameters collected at the end of the experimental phases. The data are presented as mean ± SD.

Variable/Phase		White Fluorescent (Pretreatment)		Natural Light (Baseline)		White LED vs. Natural Light (Mid-phase)	
		Control (*n* = 16)	Treatment (*n* = 18)	Control (*n* = 16)	Treatment (*n* = 18)	Control (*n* = 16)	Treatment (*n* = 18)
Body weight (kg)		692.7 ± 67.9	654.5 ± 42.1	714.2 ± 73.2	670.3 ± 44.8	716.6 ± 80	676.0 ± 49.9
Milk yield (L/d)		48.4 ± 6.7	43 ± 6.3	47.8 ± 10	48.7 ± 9.1	38.8 ± 11	38.8 ± 7.6
Experimental Period		26 September 2019		27 September 2019–28 October 2019		29 October 2019–17 November 2019	
Sunrise ^1^		05:32		05:52		06:12	
Sunset ^1^		17:32		16:58		16:40	
Natural LD ratio		12:12		11:13		10.5:13.5	
Cowshed	Illumination type	White fluorescent		Natural light		Natural light	White LED
	Dominant wavelength (nm)	545		670		670	462
	Intensity (Lux)	140		1.7		1.7	125.4
	Illumination on–off (h)	16:30–05:30		-		-	16:30–05:30
	LD ratio	24:0		11:13		10.5:13.5	24:0

^1^ Sunrise and sunset hours on the sampling day are presented as standard time and indicate the end and the beginning of civil twilight, respectively (https://mylush.net/ accessed on 9 December 2022).

## Data Availability

The data presented in this study are available on request from the corresponding author.

## Appendix A

**Table A1:** Diet composition.

Ingredients	Amount, % of Dry Matter
Corn, ground	25.1
Wheat silage	22.8
Wheat hay	12.9
Gluten feed	10.2
Dried distillers grain	7.1
Soybean meal	4.8
Rapeseed meal	4.2
Lactose water	2.6
Wheat grain, rolled	2.4
Protected fat	1.9
Cottonseed	1.5
Barley grain, rolled	1.1
Clover hay	0.9
Calcium salts	0.9
Bicarbonate	0.7
Limestone	0.5
Urea	0.3
Vitamins and minerals ^1^	
Chemical composition	
Dry matter (%)	64.6
Ether extracts (%)	4.4
Crude protein (%) Crude NDF (%) ^2^	16.5 28.7
NDF–forage (%) ^3^	17.5
NE_L_ (Mcal/gDM) ^4^	1.78

^1^ Contained (per kg of dry matter) 20,000,000 IU of vitamin A; 2,000,000 IU of vitamin D; 15,000 IU of vitamin E; 6000 mg of Mn; 6000 mg of Zn; 2000 mg of Fe; 1500 mg of Cu; 120 mg of I; 50 mg of Se; and 20 mg of Co. ^2^ NDF, Neutral detergent fiber from forage and non-forage sources. ^3^ NDF from forage sources. ^4^ NE_L_ = Diet net energy for lactation, calculated for maintenance levels, using NRC (2001) recommendations.

**Table A2:** Composition of the fatty acid standard mix (Merck, 18919-1AMP) and their detection and quantification.

Fatty Acid Standard	Fatty Acid Group	Status
Butyric Acid (C4:0)	Saturated	Quantified
Caproic Acid (C6:0)	Saturated	Quantified
Caprylic Acid (C8:0)	Saturated	Quantified
Capric Acid (C10:0)	Saturated	Quantified
Lauric Acid (C12:0)	Saturated	Quantified
Myristic Acid (C14:0)	Saturated	Quantified
Pentadecylic Acid (C15:0)	Saturated	Quantified
Palmitic Acid (C16:0)	Saturated	Quantified
Margaric Acid (C17:0)	Saturated	Quantified
Stearic Acid (C18:0)	Saturated	Quantified
Myristoleic Acid (C14:1)	Monounsaturated	Quantified
Palmitoleic Acid (C16:1)	Monounsaturated	Quantified
Oleic Acid (C18:1)	Monounsaturated	Quantified
Conjugated Linoleic Acid, CLA (C18:2)	Polyunsaturated	Quantified
Linoleic Acid (C18:2)	Polyunsaturated	Quantified
Undecanoic Acid (C11:0)	Saturated	Undetected
Tridecanoic Acid (C13:0)	Saturated	Undetected
Arachidic Acid (C20:0)	Saturated	Undetected
Heneicosanoic Acid (C21:0)	Saturated	Undetected
Behenic Acid (C22:0)	Saturated	Undetected
Tricosanoic Acid (C23:0)	Saturated	Undetected
Lignoceric Acid (C24:0)	Saturated	Undetected
Cis-10-Pentadecenoic Acid (C15:1)	Monounsaturated	Undetected
Cis-10-Heptadecenoic Acid (C17:1)	Monounsaturated	Undetected
Cis-9-Oleic Acid (C18:1)	Monounsaturated	Undetected
Cis-11-Eicosenoic Acid (C20:1)	Monounsaturated	Undetected
Erucic Acid (C22:1)	Monounsaturated	Undetected
Nervonic Acid (C24:1)	Monounsaturated	Undetected
Linolelaidic Acid (C18:2)	Polyunsaturated	Undetected
Gamma-Linolenic Acid (C18:3)	Polyunsaturated	Undetected
Linolenic Acid (C18:3)	Polyunsaturated	Undetected
Cis-11,14,17-Eicosatrienoic Acid (C20:3)	Polyunsaturated	Undetected
Cis-11,14-Eicosadienoic Acid (C20:2)	Polyunsaturated	Undetected
Cis-8,11,14-Eicosatrienoic Acid (C20:3)	Polyunsaturated	Undetected
Arachidonic Acid (C20:4)	Polyunsaturated	Undetected
Cis-5,8,11,14,17-Eicosapentaenoic Acid (C20:5)	Polyunsaturated	Undetected
Cis-13,16-Docosadienoic Acid (C22:2)	Polyunsaturated	Undetected
Cis-4,7,10,13,16,19-Docosahexaenoic Acid (C22:6)	Polyunsaturated	Undetected

**Table A3:** The proportions of total fat in whole milk (g/100 mL of milk) and of fatty acids in milk fat (g/100 g of milk fat), according to milking time and experimental phase (i.e., illumination condition).

	Baseline—Natural Light						Treatment—White LED Illumination vs. Natural Light					
	Control			Treatment			Control			Treatment		
	03:30	13:30	*p*	03:30	13:30	*p*	03:30	13:30	*p*	03:30	13:30	*p*
Fat	3.8 ± 0.4	3.1 ± 0.2	0.164	3.9 ± 0.2	3.6 ± 0.2	0.043	3.4 ± 0.2	3.9 ± 0.3	0.041	3.6 ± 0.2	4.1 ± 0.1	0.005
Saturated fatty acids	72 ± 0.7	70.3 ± 1	0.003	73 ± 1	72 ± 0.5	<0.001	71 ± 0.8	69 ± 0.8	0.003	73 ± 0.5	72 ± 0.5 #	0.206
Butyric Acid (C4:0)	6.2 ± 0.2	6.3 ± 0.2	0.842	6.4 ± 0.3	6.1 ± 0.2	0.522	7 ± 0.2 #	5.8 ± 0.2	0.001	6.2 ± 0.2	6.6 ± 0.1 $	0.083
Caproic Acid (C6:0)	3.0 ± 0.1	2.9 ± 0.1	0.663	3.2 ± 0.1	3.0 ± 0.1	0.377	3.5 ± 0.1 #	2.7 ± 0.1	<0.001	3.1 ± 0.1	3.3 ± 0.1 $	0.058
Caprylic Acid (C8:0)	1.5 ± 0.1	1.4 ± 0.1	0.02	1.7 ± 0.1	1.6 ± 0.1	0.074	1.9 ± 0.1 #	1.4 ± 0.1	<0.001	1.6 ± 0.1	1.8 ± 0.0 $	0.145
Capric Acid (C10:0)	3.2 ± 0.1	2.9 ± 0.1	0.004	3.5 ± 0.1	3.2 ± 0.1	<0.001	3.2 ± 0.1	2.8 ± 0.1	0.001	3.2 ± 0.2	3.3 ± 0.1 *	0.549
Lauric Acid (C12:0)	3.5 ± 0.1	3.3 ± 0.1	0.004	3.8 ± 0.1	3.6 ± 0.2	0.003	3.4 ± 0.1	3.2 ± 0.1	0.011	3.5 ± 0.2	3.4 ± 0.2	0.139
Myristic Acid (C14:0)	10 ± 0.1	10 ± 0.2	0.022	10 ± 0.2	10 ± 0.2	1.0	9.8 ± 0.2	9.6 ± 0.3	0.850	10 ± 0.2	9.8 ± 0.2	0.246
Pentadecylic Acid (C15:0)	1.1 ± 0.1	1.1 ± 0.1	0.058	1.0 ± 0.1	1 ± 0.1	0.874	1.1 ± 0.0	1.0 ± 0.1	0.112	1.0 ± 0.0	1.0 ± 0.0	0.34
Palmitic Acid (C16:0)	32.2 ± 0.5	31.0 ± 0.4	0.008	32 ± 0.5	32 ± 0.5	0.046	31.8 ± 0.5	32.2 ± 0.5	0.43	33 ± 0.5 #	32.5 ± 0.4	0.015
Margaric Acid (C17:0)	0.6 ± 0.0	0.6 ± 0.0	0.166	0.7 ± 0.1	0.6 ± 0.0	0.666	0.7 ± 0.0	0.6 ± 0.0	0.003	0.7 ± 0.1	0.6 ± 0.0	0.957
Stearic Acid (C18:0)	10.2 ± 0.3	10.0 ± 0.2	0.141	11 ± 0.3	11 ± 0.3	0.812	9.1 ± 0.2	9.8 ± 0.3	0.001	10 ± 0.3 #	9.8 ± 0.3	0.08
Monounsaturated fatty acids	23.7 ± 0.6	25 ± 0.7	0.031	23 ± 0.4	24 ± 1	0.001	24.3 ± 0.7 *	26.7 ± 0.9 #	0.003	23 ± 0.4	23.8 ± 0.4	0.122
Myristoleic Acid (C14:1)	1.0 ± 0.1	1.0 ± 0.1	0.009	1.0 ± 0.1	1.0 ± 0.1	0.02	1.1 ± 0.1	1.2 ± 0.1	0.325	1.1 ± 0.1	1.1 ± 0.1	0.222
Palmitoleic Acid (C16:1)	1.7 ± 0.1	1.8 ± 0.1	0.394	1.6 ± 0.1	1.6 ± 0.1	0.831	2.0 ± 0.2	2.0 ± 0.2	0.430	1.7 ± 0.1	1.8 ± 0.1	0.186
Oleic Acid (C18:1)	21.1 ± 0.6	22 ± 0.6	0.05	20 ± 0.4	21 ± 0.4	0.003	21.1 ± 0.6	2.4 ± 1 #	0.003	20 ± 0.4	20.9 ± 0.4	0.199
Polyunsaturated fatty acids	4.5 ± 0.2	5 ± 0.2	0.002	4.2 ± 0.1	4.4 ± 0.1	0.011	4.3 ± 0.1	4.3 ± 0.3	0.21	4.1 ± 0.1	4.2 ± 0.1	0.185
Linoleic Acid (C18:2)	3.7 ± 0.1 *	4.1 ± 0.2 *	0.002	3.5 ± 0.1	3.6 ± 0.1	0.03	3.6 ± 0.1	3.6 ± 0.3	0.258	3.4 ± 0.1	3.5 ± 0.1	0.166
Conjugated Linoleic Acid, (C18:2)	0.7 ± 0.0	0.8 ± 0.1 *	0.004	0.6 ± 0.0	0.7 ± 0	0.011	0.7 ± 0.0	0.8 ± 0.1	0.043	0.7 ± 0.0	0.7 ± 0.0	0.366

The data are presented as mean ± SE. The Wilcoxon non-parametric test was used to assess the effect of milking time in each group. The Mann–Whitney non-parametric test was used to assess differences between the control and treatment groups, for the milking time at each experimental period. *, *p* < 0.05; #, *p* < 0.01, $, *p* < 0.001 between treatments within milking hours, by the Mann–Whitney non-parametric test.
